# Supplementary material for: Assessment of knowledge and practice of breast self-examination among reproductive age women in Akatsi South district of Volta region of Ghana
Source: PLoS One. 2019 Dec 30;14(12):e0226925. doi: 10.1371/journal.pone.0226925 (PMC6936838; doi:10.1371/journal.pone.0226925)
Supplement: Attachment — Submitted filename: Rebuttal Letter BSE.docx [file pone.0226925.s001.docx]

Department of Family and Community Health

School of Public Health

University of Health and Allied Sciences

Hohoe-Ghana

11/4/19

Dear Editor,

**REBUTTAL LETTER TO REVIEWERS COMMENTS**

I am humbled to submit this letter describing how the comments of the reviewers to our manuscript entitled‘*Assessment of Knowledge And Practice Of Breast Self-Examination Among Reproductive Age Women in Akatsi South District of Volta Region of Ghana.* We have addressed all comments by the reviewers in the manuscript and describe below how those comments were addressed and where in the manuscript to find the changes effected.

We hope that addressing the comments to the best way we possibly could do will make our manuscript more meritorious for acceptance and publication. We count on your usual cooperation. Thank you.

Awolu Adam

**Comments:**

Abstract:

1. Distinct or township name has been replaced with Akatsi South District in Volta Region of Ghana
2. The methodology in the abstract has been updated to include variables that were measured. The age range of 15-45 was a typing error and the correct range of 15-49 has been indicated.
3. The statement is reworded to state ‘key strategy to early detection of breast cancer as recommended.

Introduction:

4. After reading the introduction again, we realized the first two sentences did were not needed and so they are deleted.
5. The introduction has been cut down and aligned well as recommended.
6. Korle-Bu Teaching Hospital and Volta region are both in Ghana and that have been clarified in the manuscript on page 4 and in bold.
7. The heading ‘awareness of breast self-examination and breast cancer’ has been removed as recommended.
8. The spelling of ‘Resource’ has been corrected.
9. A reference has provided for the statement “Breast self-examination is critical in resource limited settings as a strategy to reducing mortality due to late reporting and diagnosis” on page 5.
10. A reference has been added to the statement “Breast self-exam (BSE), or regularly examining your breasts on your own, can be an important way to find a breast cancer early, when it's more likely to be treated successfully’’ on page 5.
11. A reference has been added “Breast self-examination as a screening tool for breast cancer in developing countries is advocated in view of its cost-effectiveness. This is on page 5.
12. The research objective has been rewritten to reflect exploring variables or characteristics that are associated with knowledge of and practice breast self-examination. This is on page 6 in bold.

Methods:

13. Details about the study setting have been added including the population, rural, and the district all in the Volta Region of Ghana. The details are pages 6 in bold.
14. The justification of inclusion of women ages 15-49 is provided on page 7 in bold.
15. Justification of including only women who have been resident in Akatsi South district for 5 years is provided on page 7 in bold.
16. The variables that were measured in the study including demographic, knowledge, and breast self-examination practice variables have been enumerated on page 7 in bold.
17. Descriptive statistics and chai square analysis were computed and these have been stated in the methods section on page 7.

Results:

18. “These socio-demographic variables were crucial in understanding the responses of the participants and the findings in this study.” This statement has been moved to discussion section as recommended. This is on page 17 in bold.
19. As recommended, the statement “To measure awareness of breast cancer among the participants, two questions were included in the questionnaire asking whether or not they had heard of breast cancer as a disease and whether or not they considered breast cancer as a common disease among women in Ghana.” is moved to methods section. This is on page 7 in bold.
20. ‘Refusal to breastfeed’ has been substituted with ‘did not breastfeed’ and this is on page 9 and in bold
21. A statement has been made to confirm that answers provided by the women and listed here were correct and this is on page 10 in bold.
22. “After the knowledge of breast cancer was determined above as to either good or poor knowledge, it was important to find out the demographic characteristics of respondents that may have influenced their level of knowledge.” This statement has been replaced the statement ‘**Association between the demographic variables and knowledge of breast cancer was also measured using *X^2^’*** and it is the methods section on page 7.
23. The title of table 2 is updated to read **Demographic Variables and Their Association to knowledge of breast cancer.** This is on page 12.
24. “Even though those who reported not practicing BSE gave various reasons for not practicing, we wanted to statistically find out the predictors of BSE in this sample and so a chi square (X 2 ) test of significance was performed.” This is replaced by ‘’**Besides reasons provided by those who did not practice BSE, we performed X^2^ test of significance to determine significant predictors of BSE practice’’** and in the methods section**.**

Discussion:

25. The meaning of market women has been clarified on page 15 in bold.
26. We have added a statement explaining our view on the association between age and breast self-examination. This is on pages 16 and 17 in bold.
27. Limitations and strengths have been added to the discussion section on pages 17 and 18 in bold.
28. There is a statement in the conclusion part suggesting public health practice implication from the findings of this study. This in bold on page 18.
