# Supplementary material for: Assessment of knowledge and practice of breast self-examination among reproductive age women in Akatsi South district of Volta region of Ghana
Source: PLoS One. 2019 Dec 30;14(12):e0226925. doi: 10.1371/journal.pone.0226925 (PMC6936838; doi:10.1371/journal.pone.0226925)
Supplement: Attachment — Submitted filename: Response To Reviewers BSE.docx [file pone.0226925.s002.docx]

11/27/19

Dear Sir/Madam,

**RESPONSE TO REVIEWERS COMMENTS:**

I humbly present this letter to provide responses to comments made by reviewers after review of our manuscript ‘*Assessment of Knowledge And Practice Of Breast Self-Examination Among Reproductive Age Women in Akatsi South District of Volta Region of Ghana’*. We looked at each of the comments made by the two reviewers and addressed each comment accordingly. Our responses and actions we took are presented below.

Reviewer #1: Introduction

1. Authors should refer to GLOBOCAN 2012 as reference 1. As the manuscript Ghartey et al refers to Ghanaian specific breast cancer distribution.

**Response**: *Ferlay J, Soerjomataram I, Dikshit R, Eser S, Mathers C, Rebelo M, Parkin DM, Forman D, Bray F. Cancer incidence and mortality worldwide: sources, methods and major patterns in GLOBOCAN 2012. International journal of cancer. 2015 Mar 1;136(5):E359-86.*

The above reference has been provided as reference. Ferlay and colleagues worked on GLOBOCAN 2012 and is an appropriate reference on GLOBOCAN 2012.

2. The section on BSE of the introduction is repetitive and verbose. It would serve the authors to reduce the length and be more concise.

**Response**: The BSE section has been cut down and well synchronized.

3. Methods: Need to be listed in methods:

A summary of the questions asked, which language or languages (s) etc. Were the questions on scales, yes/no, categorical. What variables were collected from study participants. Where were the study participants identified?

**Response:** The summary of the questions included breast cancer and breast self-examination knowledge and BSE practice. The variables collected from the participants are clarified in the methods section on pages 7 and 8 and lines 148 to 155.

4. Results: Is the entire region rural or are there more urban centers within the region? Can the authors analyze based on this category.

**Response**: Except for Ho, the capital town of the Volta Region of Ghana, which is urban, the rest of the region is predominantly rural. Akatsi South district is predominantly rural and only women from the district were recruited into the study. Therefore, the data analysis reflects predominantly rural women and we do not have urban data to analyze in this study.

Reviewer #2: This is a timely article. The topic is good and the data connect to the discussion at hand. It is appreciated that the significant and non-significant findings are presented. Edits would include using past tense throughout and clarifying some of the statements. I have uploaded a scanned copy of my comments.

**Response:**

All the edits recommended by the reviewer 2 have been effected throughout the manuscript. We did not feel the need to track all the edits with track changes.
